# Pain control based on oscillatory brain activity using transcranial alternating current stimulation: An integrative review

**DOI:** 10.3389/fnhum.2023.941979

**Published:** 2023-01-19

**Authors:** Naoyuki Takeuchi

**Affiliations:** Department of Physical Therapy, Akita University Graduate School of Health Sciences, Akita, Japan

**Keywords:** brain communication, chronic pain, interpersonal interaction, oscillatory brain activity, transcranial alternating current stimulation

## Abstract

Developing effective tools and strategies to relieve chronic pain is a high-priority scientific and clinical goal. In particular, the brain regions related to pain processing have been investigated as potential targets to relieve pain by non-invasive brain stimulation (NIBS). In addition to elucidating the relationship between pain and oscillatory brain activity, transcranial alternating current stimulation (tACS), which can non-invasively entrain oscillatory brain activity and modulate oscillatory brain communication, has attracted scientific attention as a possible technique to control pain. This review focuses on the use of tACS to relieve pain through the manipulation of oscillatory brain activity and its potential clinical applications. Several studies have reported that tACS on a single brain reduces pain by normalizing abnormal oscillatory brain activity in patients with chronic pain. Interpersonal tACS approaches based on inter-brain synchrony to manipulate inter-brain communication may result in pain relief *via* prosocial effects. Pain is encoded by the spatiotemporal neural communication that represents the integration of cognitive, emotional-affective, and sensorimotor aspects of pain. Therefore, future studies should seek to identify the pathological oscillatory brain communication in chronic pain as a therapeutic target for tACS. In conclusion, tACS could be effective for re-establishing oscillatory brain activity and assisting social interaction, and it might help develop novel approaches for pain control.

## Introduction

The brain regions involved in pain processing include the primary and secondary somatosensory cortices; primary motor and supplementary motor cortices; insular cortex; anterior cingulate cortex; thalamus; regions within the prefrontal and parietal cortices; and regions involved in emotion, memory, and fear processing in the amygdala, hippocampus, and subcortical structures, including the basal ganglia (Martucci and Mackey, 2018; Geuter et al., 2020). Among these brain regions, cortical sites near the scalp have been investigated as potential targets to reduce pain by non-invasive brain stimulation (NIBS), which can alter cortical excitability through approaches, such as repetitive transcranial magnetic stimulation and transcranial direct current stimulation (O’Connell et al., 2018; Kandiæ et al., 2021).

Neurophysiological studies using electroencephalography (EEG) and magnetoencephalography (MEG) have demonstrated that neural oscillatory frequencies corresponding to the theta (4–7 Hz), alpha (8–12 Hz), beta (13–29 Hz), and gamma bands (30–100 Hz) are associated with pain processing (Ploner et al., 2017). The elucidation of neural oscillations is not only helpful for the diagnosis and classification of chronic pain but might also help develop a target for therapeutic strategies. Transcranial alternating current stimulation (tACS), which can non-invasively modulate oscillatory brain activity, has recently attracted scientific attention as a promising technique for pain control by targeting neural oscillations. Interestingly, tACS is a non-invasive method that is used to entrain neuronal activity into certain frequency patterns *via* the application of a weak oscillatory current to the brain through the scalp (Helfrich et al., 2014; Vosskuhl et al., 2018). It shifts the neural membrane potential from its resting potential toward slightly more depolarized or hyperpolarized states. Depolarized neurons are more likely to fire in response to other neurons. This is a potential mechanism by which tACS can entrain neural oscillations time-locked to the frequency of the weak stimulation (Fertonani and Miniussi, 2017; Liu et al., 2018).

As a mechanism of the brain’s complex processes based on neural communication across distant brain areas, synchronization of oscillatory brain activity among networks is hypothesized to facilitate information transfer across brain regions by temporally aligning neural processing (Fries, 2015). Therefore, synchronous tACS over distant regions can increase functional connectivity between the targeted brain regions by synchronously entraining brain oscillations. Contrastingly, desynchronous tACS has the opposite effect (Vosskuhl et al., 2018; Cabral-Calderin and Wilke, 2020). Considering the hypothesis that pain is encoded by the spatiotemporal brain network communication that represents the integration of cognitive, emotional-affective, and sensorimotor aspects of pain (Kucyi and Davis, 2015; Ploner et al., 2017), applying tACS between distant brain regions might help control the progress of pain chronicity by modulating the pathological oscillatory brain communication.

This review explores the potential for applying tACS techniques to achieve pain control by modulating oscillatory brain activity. First, this review focuses on tACS studies that target a single brain for pain control by normalizing oscillatory brain activity. Next, inter-brain synchrony related to pain processing in the context of social interaction revealed by hyperscanning, which is a neuroimaging technique used to measure the activity of multiple brains simultaneously, is reviewed. Then, this review discusses a potential dual-brain approach using tACS to reduce pain *via* prosocial effects. This concurrent manipulation of brain activities helps elucidate the role of inter-brain oscillatory communication in pain processing *via* interpersonal interaction. However, the application of dual-brain stimulation to modulate the clinician’s brain activity in clinical settings is unrealistic. Therefore, this review discusses the potential of using brain-to-brain interfaces, which allow two brains to mutually exchange decoded neural information for analgesia *via* interpersonal interaction. Finally, to inform future pain control research, the altered functional connectivity among large-scale distributed brain networks is reviewed and the potential of tACS approaches to modulate network-level abnormalities of oscillatory communication in chronic pain is discussed.

## Pain control targeting abnormal neural oscillations

Accumulating evidence shows that chronic pain is closely associated with altered neural oscillations (Ploner et al., 2017). The most noticeable change in patients with chronic pain is an increase in theta oscillations (Pinheiro et al., 2016), a phenomenon that is explained by the thalamocortical dysrhythmia model of chronic pain (Llinás et al., 2005; Vanneste et al., 2018). In this model, abnormal nociceptive input induces thalamic theta oscillations, which in turn entrains thalamocortical loops. At the cortical level, the abnormal theta oscillations induce disinhibition of neighboring areas, which might result in abnormal gamma oscillations. Abnormal alpha oscillations are also well-known changes associated with chronic pain. Systematic reviews have reported that low-alpha oscillations are increased in patients with chronic pain (Pinheiro et al., 2016; Mussigmann et al., 2022). Furthermore, beta oscillations are reported to increase during chronic pain (Lim et al., 2016; May et al., 2019). However, abnormality in these oscillations have not always been observed in patients with chronic pain (Schmidt et al., 2012), and seemingly conflicting results that the suppression of alpha oscillations is correlated with pain severity have also been reported (Camfferman et al., 2017). Therefore, neural oscillatory modulation using tACS can investigate the relationship between pain and specific frequency patterns across different pathologies and may consequently be used as a promising therapeutic tool for chronic pain.

A literature search regarding original research using tACS for pain control was carried out in PubMed, Scopus, and Web of Science using the following search terms: (“tACS” OR “transcranial alternative current stimulation”) AND (“pain relief” OR “pain control” OR “reduce pain” OR “modulate pain” OR “analgesia”). Searches were limited to papers published in English before April 2022. No inclusion or exclusion criteria were established because there were only five studies that modulated experimental and chronic pain using tACS as a result of a literature search. The effects of tACS on experimental pressure pain in healthy individuals were first investigated by Arendsen et al. (2018). The authors showed that alpha-tACS over bilateral sensorimotor cortices can reduce subject-reported pain when the intensity of an upcoming pain was uncertain. However, another study reported that alpha-tACS over bilateral sensorimotor cortices had no effect on experimental heat pain in healthy participants (May et al., 2021). Clinical studies have investigated whether tACS modulates pain intensity in patients with chronic low back pain. One study reported that alpha-tACS over bilateral sensorimotor cortices reduced pain intensity compared to the sham condition (Ahn et al., 2019). Moreover, increasing alpha oscillations after tACS positively correlated with the degree of pain reduction, indicating a relationship between pathological reduced alpha oscillations and chronic pain. Another study performed alpha-tACS over bilateral sensorimotor cortices in patients with chronic low back pain (Prim et al., 2019), and the results showed that twice as many responders of pain reduction were in the tACS groups compared with the sham condition.

Few tACS studies have adapted conditions other than somatosensory alpha oscillations to modulate pain. A recent study investigated whether gamma-tACS over bilateral prefrontal cortices modulates experimental heat pain (May et al., 2021). Gamma oscillations in prefrontal areas encode pain intensity during experimental heat pain in healthy participants (Schulz et al., 2015; Nickel et al., 2017) and are associated with ongoing back pain intensity (May et al., 2019). However, gamma-tACS over bilateral prefrontal cortices had no effect on pain intensity in healthy participants. Another recent study investigated whether the normalization of abnormal brain oscillations by delivering personalized tACS reduces pain in patients with fibromyalgia syndrome (Bernardi et al., 2021). Patients showing spectral power corresponding to higher slow frequencies (1–10 Hz) were stimulated with beta-tACS at 30 Hz, while those showing higher fast frequencies (10.5–30 Hz) were stimulated with theta-tACS at 4 Hz. The primary motor cortex (M1) and sensorimotor areas were stimulated as the main targets based on the topography of EEG abnormalities. The authors showed that personalized tACS combined with a physical program increased alpha power and reduced patient-reported pain.

Thus, alpha-tACS over the sensorimotor cortex may represent a potential approach to relieve chronic pain. However, tACS studies for pain control are limited, and this hypothesis is strongly influenced by publication and reporting biases. Moreover, the alpha-tACS effect on experimental pain in healthy individuals is inconsistent. The personalized frequency-location parameters might reduce variability in the tACS response. Moreover, important insights into the neural mechanisms of chronic pain could be yielded using appropriate tACS parameters considering pathological factors, such as disease and duration of onset. So far, I have discussed tACS approaches targeting a single brain. Next, the focus will shift to tACS based on inter-brain communication between two brains in the context of interpersonal interactions.

## Pain control targeting inter-brain oscillatory communication

It is known that interpersonal interactions relieve pain (Jensen et al., 2014; Shamay-Tsoory and Eisenberger, 2021). To elucidate the neural mechanism of analgesia *via* interpersonal interaction, a neuroimaging method that allows simultaneous investigation in multiple persons is useful. Studies measuring the activity of multiple brains simultaneously, termed hyperscanning, have revealed that various interpersonal factors can be reflected in inter-brain communication between individuals engaging in joint actions and communication (Dai et al., 2018; Pan et al., 2020). Hyperscanning studies seeking to elucidate the neural mechanism of pain processing have targeted mainly pain empathy, interpersonal touch, and clinician-patient relationships.

Pain empathy enables one to understand or recognize others’ pain perception by observing them experiencing pain, inducing emotional sharing associated with pain reduction (Fitzgibbon et al., 2010; Jensen et al., 2014). Peng et al. (2021) performed EEG hyperscanning to assess the neuronal mechanism of pain empathy during a pain-sharing task wherein electrical pain stimulation was delivered to one participant of a dyad; expecting high-intensity pain induced greater alpha-band inter-brain synchrony of sensorimotor areas between pain-takers and pain-observers than did expecting low-intensity pain. Moreover, mediation analysis indicated that sharing a painful experience induces prosocial behavior within dyads through alpha-band inter-brain synchrony. Interpersonal touch has an analgesic effect on newborns (Gray et al., 2000), patients with cancer (Aghabati et al., 2010), and patients with chronic pain (Smith et al., 2009). Goldstein et al. (2018) performed EEG hyperscanning of romantic partners to examine the association between oscillatory brain communication and analgesia during hand-holding. They showed that hand-holding increases the alpha-band inter-brain synchrony of central regions during heat pain stimulation. Moreover, alpha-band inter-brain synchrony correlates with the magnitude of analgesia and the observer’s empathic accuracy. A good clinician-patient relationship has a positive effect on patient outcomes and accounts for a substantial part of psychological pain reduction (Kaptchuk and Miller, 2015; Mistiaen et al., 2016). Using functional magnetic resonance imaging (fMRI), Ellingsen et al. (2020) investigated the brain activity concordance when clinicians treated patients with chronic pain. They found that patient-clinician dyads that had established a therapeutic alliance, relative to the control group, showed increased dynamic concordance between dyads in brain networks related to theory-of-mind and social mirroring processing.

Neural coupling during interpersonal interaction may either cause or be caused by behavioral entrainment, emotional sharing, and common understanding, and these factors may work jointly to promote analgesia (Koole and Tschacher, 2016; Kingsbury and Hong, 2020). Therefore, artificial induction of inter-brain synchrony has the potential to promote analgesia through prosocial effects. Currently, tACS is the main type of NIBS utilized for dual-brain stimulation to induce inter-brain synchrony in human studies (Takeuchi, 2022). A previous study evaluated whether tACS applied simultaneously to two individuals (hyper-tACS) alters interpersonal interaction (Novembre et al., 2017). Synchronous beta-tACS over M1 in dyads with both individuals performing a finger-tapping task enhanced interpersonal movement synchrony, compared with desynchronous or sham stimulation. Phase coupling of brain oscillations across two individuals’ M1s supports the interpersonal alignment of sensorimotor processes that regulate rhythmic action, thereby facilitating synchronous interpersonal movement. It has been reported that the higher the movement synchronization between the patient and clinician, the lower the patient-reported pain to heat stimulation administered by the clinician, and the higher the trust in the clinician (Goldstein et al., 2020). Therefore, beta-band hyper-tACS over M1 might promote the therapeutic alliance *via* movement synchrony between the clinician and patient, providing a good foundation for facilitating pain therapy.

As aforementioned, alpha-band inter-brain synchrony between dyads is involved in pain empathy and interpersonal touch processing. Although it remains unclear whether the specific frequency and site of inter-brain communication are associated with the clinician-patient relationship, it is well known that the alpha–mu band in centroparietal areas comprises the most robust oscillations of inter-brain synchrony during social interaction (Tognoli and Kelso, 2015). Therefore, alpha-band hyper-tACS over centroparietal areas is a potential candidate for the induction of analgesia *via* prosocial interaction. However, applying NIBS to the clinician’s brain is not realistic in the clinical field because of ethical issues regarding the manipulation of healthy brains. Thus, rather than being a therapeutic tool, hyper-tACS is useful as a technique to investigate the role of frequency and site-specific brain-to-brain communication in analgesia *via* interpersonal interaction, which can complement hyperscanning and single-brain stimulation studies. After clarifying the relationship between inter-brain oscillatory communication and analgesia, it is desirable to use the brain-to-brain interface, which could induce inter-brain synchrony by modulating the patient’s brain activity using tACS and adjusting it to the clinician’s brain activity. A brain-to-brain interface enables the mutual exchange of decoded neural information between two brains through a brain-computer interface. This interface receives the sender’s neural information and transmits it to the receiver’s brain *via* electrical stimulation (Nam et al., 2021). Direct information transmission from the brain of a clinician to that of a patient using a brain-to-brain interface could facilitate interpersonal interaction and more complex bidirectional clinician-patient interactions to achieve pain control.

## Future direction of tACS research for pain control

Pain is a complex, multifaceted experience that has physiological, emotional-affective, and cognitive dimensions, indicating that chronic pain progression might be influenced by altered integration between sensory and contextual process networks (Kucyi and Davis, 2015; Ploner et al., 2017). Consistent with this hypothesis, neuroimaging studies have reported that functional connectivity changes in chronic pain are widespread and involve the sensorimotor network (SMN) as well as self-reflection (default mode, DMN), cognitive control (frontal-parietal, FPN), and emotion (salience, SN) networks (Farmer et al., 2012; Hemington et al., 2018; Ellingsen et al., 2021). The DMN, which is associated with self-referential processing and theory-of-mind, is consistently disturbed in patients with chronic pain (Loggia et al., 2013; Baliki et al., 2014). In contrast, the FPN is involved in the cognitive control of behavior. Kutch et al. (2017) showed that strong functional connectivity within the FPN was associated with the improvement of chronic pain after 3 months. The abnormal connectivity within SN, which is involved in emotional control and social behavior regarding the detection of salient stimuli, has been reported in patients with chronic pain (Borsook et al., 2013). Moreover, while each network independently demonstrates changes in functional connectivity, it has also been reported that hyper-connectivity between these networks exists in patients with chronic pain (Napadow et al., 2010; Hemington et al., 2018; van Ettinger-Veenstra et al., 2019; Ellingsen et al., 2021). Consistent with these findings, clinical studies have reported that pharmacological pain therapy reduced functional connectivity between DMN-SN and DMN-SMN (Rogachov et al., 2019), and that cognitive-behavioral pain therapy reduced the functional connectivity between DMN, FPN, and SN in patients with chronic pain (Meier et al., 2020). The neural oscillatory coupling between distant brain regions serves the integrative functions by facilitating information flow throughout the brain (Fries, 2015). Therefore, the analysis of neural oscillatory communication in chronic pain might contribute to the development of pain control targets of tACS to help modulate the abnormal functional connectivity. However, the altered functional connectivity of chronic pain, as aforementioned, were observed using resting-state fMRI and fluctuations of blood oxygenation level-dependent signals below 0.1 Hz. Recent EEG/MEG studies have investigated the change of fine neural oscillatory communication within and between these networks in chronic pain (Choe et al., 2018; Kim et al., 2020; Kisler et al., 2020; Heitmann et al., 2021). In contrast to fMRI, MEG studies have demonstrated reduced functional connectivity within and between networks across multiple frequency bands in patients with chronic pain (Choe et al., 2018; Kim et al., 2020). Moreover, the improvement of pain intensity after 6 months interdisciplinary multimodal pain therapy was associated with an increase in the global network at theta frequencies (Heitmann et al., 2021). Thus, the change in fine neural oscillatory communication has been gradually reported in chronic pain, but an overarching framework remains unclear and requires further investigation concerning the therapeutic target of tACS.

Further investigation with regard to tACS approaches is warranted in future studies. Networks, such as the DMN and SN, comprise deep brain sites that are difficult to stimulate with conventional NIBS. As a new tACS technique, transcranial temporal interference stimulation may be able to solve this problem (Grossman et al., 2017). This technique has been proposed to stimulate deep brain regions with specific frequencies and amplitudes using temporally interfering electric fields, although it needs validation of safety in humans. Furthermore, identifying optimal tACS parameters in the future is essential with regard to achieving pain control. Personalized tACS according to the brain state by closed-loop systems, comprising EEG/MEG combined with tACS, is desirable to stabilize and promote pain reduction. Moreover, this closed-loop system might be able to appropriately set the tACS parameters based on the disease-specific frequency and site in pathological conditions that are different from those of healthy participants. However, further systematic testing is required to analyze the oscillatory brain activities in real-time and remove the artifacts produced by tACS without removing a substantial amount of valuable electrophysiological signals (Thut et al., 2017; Zarubin et al., 2020). Finally, it should be noted that tACS-induced entrainment on neural oscillations cannot explain all longer-lasting aftereffects of tACS although they are related (Veniero et al., 2015; Geffen et al., 2021). A detailed elucidation of the neuroplastic tACS effect is requisite for its clinical application to plastic changes in chronic pain.

In conclusion, despite the translational potential of tACS based on oscillatory brain activity for pain control, the methodology remains in its infancy and poses several issues that require greater innovation. Neuroscientific knowledge of intra- and inter-brain communication related to pain processing is instrumental in developing optimal tACS approaches as non-pharmaceutical alternatives for the treatment of chronic pain.

## Author contributions

The author confirms being the sole contributor of this work and has approved it for publication.
